# Accumulation of lifestyle and psychosocial problems and persistence of adverse lifestyle over two-year follow-up among Finnish adolescents

**DOI:** 10.1186/1471-2458-14-542

**Published:** 2014-06-01

**Authors:** Eveliina Heikkala, Jouko Remes, Markus Paananen, Simo Taimela, Juha Auvinen, Jaro Karppinen

**Affiliations:** 1 Medical Research Center Oulu, Oulu University Hospital and University of Oulu, Aapistie 1, 90220 Oulu, Finland; 2 Finnish Institute of Occupational Health, Statistics and Health Economics, Aapistie 1, 90220 Oulu, Finland; 3 Department of Public Health, University of Helsinki, Helsinki, Finland; 4 Institute of Health Sciences, University of Oulu, Aapistie 5 B, 90220 Oulu, Finland; 5 Finnish Institute of Occupational Health, Health and Work Ability, and Disability Prevention Centre, Aapistie 1, 90220 Oulu, Finland

**Keywords:** Adolescent, Health behavior, Mental health, Cohort studies, Latent class

## Abstract

**Background:**

Adolescent’psychosocial problems associate with unhealthy behaviors, but data on co-occurring patterns is sparse. We investigated 1) whether adolescents could be categorized into meaningful subgroups with respect to psychosocial and lifestyle factors, 2) whether the prevalence of physical inactivity, overweight and smoking vary within the subgroups and 3) whether these unhealthy behaviors persist in a two-year follow-up.

**Methods:**

The study was based on a subgroup of the 1986 Northern Finland Birth Cohort, which consisted of adolescents who replied to a postal questionnaire at 16 years (n = 6792) and a subgroup of this sample at 18 years (n = 1552). Latent class analysis (LCA) was performed to establish clusters at 16 years.

**Results:**

Smoking co-existed with emotional and behavioral problems in both genders. Boys with the most inactive lifestyle slept poorly, whereas multiple problems co-occurred among girls. Those with a high body mass index (BMI) separated as groups of their own. Different combinations of adverse lifestyle and emotional and behavioral problems were relatively common in both sexes as only 51% of boys and 67% of girls belonged to the reference cluster with low probability for these findings. Physical inactivity, high BMI and smoking tended to persist over the two-year follow-up.

**Conclusions:**

It seems that lifestyle and psychosocial factors divide adolescents into distinct subgroups in which unhealthy lifestyle patterns remain between the ages of 16 and 18. This may indicate problems in other life areas and expose them to an increased risk of future health problems.

## Background

A higher proportion of adolescents in Western countries today suffer from emotional [1,2] and behavioral problems [2,3] and are overweight or obese [4,5] than earlier. In addition, the vast majority of adolescents fail to meet the recommended sufficient levels of physical activity [6,7], engage in sedentary behavior for longer than is recommended [7], and have insufficient sleeping times [8]. Both health-related behaviors [9-12] and psychological problems [13,14] influence present and later health negatively, and tend to continue from adolescence into adulthood [15-18].

A number of adolescent’lifestyle and psychosocial factors interrelate rather than have separate effects. For instance, associations between physical inactivity, sedentary behavior, poor sleeping time, body mass index, smoking [7,8,19-21], and emotional and behavioral problems [22], as well as between both lifestyle and psychosocial dimensions have been characterized [23-25]. Even though some of these relations, e.g. the association between physical inactivity and sedentary behavior [7], are well-established, most links tend to be complex, with knowledge regarding the co-occurrence of psychosocial and lifestyle factors being limited.

Recent research has utilized various statistical methods for studying the interplays of several factors, of which latent class analytic approaches has been proposed to be among the most promising [26,27]. Latent class analysis (LCA) is a statistical technique to seek uncovered groups of individuals that are similar to each other within a certain cluster on the basis of the patterns of included variables. In latent models, the distribution of participants into meaningful subgroups is based on probabilities and no assumptions of linear relationships, normal distributions, or homogeneity are required [28]. Furthermore, this model has formal fit indices to determine the optimal number of clusters [28,29].

The merits of using LCA to study the clustering of lifestyle and psychosocial factors have been illustrated in several studies conducted among adolescents and young adults [30-35]. However, most of the recent works have applied LCA to the patterning of a limited number of factors without taking into account the psychosocial and lifestyle perspectives simultaneously. We investigated how lifestyle factors (physical activity, sedentary behavior, smoking, sleeping, and overweight/obesity) and psychosocial symptoms are grouped in adolescence, and analyzed the changes in the proportions of adolescents who were inactive, overweight, and smokers within the clusters in a two-year follow-up. These unhealthy behaviors were only included as we did not have follow-up data of other factors. Given the previous findings of gender-related differences in engagement in these factors in the study population of the present work [7,36] and in other populations (e.g. [19]), boys and girls were analyzed separately.

## Methods

### Study population

The study population belongs to the 1986 Northern Finland Birth Cohort (NFBC 1986), which consists of all live-born children with an expected date of birth between 1st July 1985 and 30th June 1986 (n = 9479) in the two northernmost provinces of Finland (Oulu and Lapland). From May 2001 through April 2002, a questionnaire was sent to all living members of the cohort (n = 9215). A total of 3302 boys and 3590 girls provided information on both psychosocial and lifestyle factors studied. In 2003–2004, the follow-up data were collected, after a subgroup of this birth cohort (n = 2969), living within 100 km of the city of Oulu (Oulu back study, OBS), received a second postal questionnaire. A total of 2012 participants, with a mean age of 18, replied. Of these, 1552 (681 boys and 871 girls) had also responded to the first questionnaire. The study was approved by The Ethics Committee of the University Hospital of Oulu, and followed the principles of the Declaration of Helsinki. The participants, whose personal information was replaced by ID codes, took part on a voluntary basis and signed their informed consent, which was also obtained from their parents. The data were handled on a group level only.

### Variables used in clustering

The factors used for describing lifestyle at 16 years were level of physical activity, sleeping, smoking, sedentary behavior, and overweight/obesity, whereas internalizing and externalizing behavior represented participant’psychosocial symptoms.

We first tried to consider all variables as continuous in the latent class model to avoid loss of information, but the group sizes were too small (<5% of participants [37]) and the optimal number of subgroups, recommended by the statistical fit indices introduced in the next section, would have been more than that of the factors included. Next, we tried different combinations of continuous and categorical variables. Finally, we observed that the categorization of variables that best represent our data were the dichotomization of externalizing and internalizing problems into problem and normal range [38]; the trichotomization of physical activity level [25]; sleeping time [39,40], smoking [41], and sedentary activity level among boys [11] on the basis of recommendations or previous studies; and the use of the sedentary activity variable among girls and BMI variable among both genders as continuous variables.

Dichotomizing the variables instead of trichotomizing them would have led to information loss, especially in the case of physical activity and sedentary behavior among boys. Moreover, a dichotomization of sleeping time would have complicated the interpretability, i.e. it would have been difficult to determine whether sleeping more or less than the recommended 8–9 hours was the worst. Instead, categorizing internalizing and externalizing syndromes into problem and normal ranges was justifiable due to the clinical perspective and for easier interpretation of the results.

#### Lifestyle variables

The adolescent’participation in moderate-to-vigorous physical activity (MVPA) was elicited by asking how many hours they spent on physical activity causing at least some sweating and shortness of breath outside school hours [7]. We classified physical activity into three categories: (1) active (more than three hours of MVPA per week), (2) moderately active (2–3 hours of MVPA per week), and (3) inactive (1 hour or less of MVPA per week). Sleeping time was elicited with the question: “How many hours on average do you sleep per day?” A sleeping time of 8.5 to 9.2 hours is regarded as optimal in adolescence [39,40]. Therefore sleeping was categorized as: (1) less than 8 hours, (2) 8–9 hours, and (3) more than 9 hours per day. Information on smoking was obtained with the questions: “Have you ever smoked?”, “Have you ever smoked regularly in your life?” and “How much do you currently smoke? [10]. On the basis of the responses, the participants were divided into three groups: (1) non-smokers, (2) 0.1–1.0 pack-years by the age of 16 years, and (3) over 1.0 pack-year, where one pack-year is equivalent to 15 cigarettes smoked per day for a year. To elicit the adolescent’average sitting time per day, we asked about their participation in four different sedentary activities (watching television, reading books or magazines, playing or working on a computer, and other sedentary activities) outside school hours. The adolescents reported hours per day for each of these four activities. We then summed up all sedentary hours, and used a continuous variable for girls, and categorized the total number of sedentary hours per day for boys to: (1) 4 hours or less per day, (2) 4.1–7.9 hours, and (3) 8 hours or more per day [9]. Body weight and height were obtained through a health examination (n = 6068, participation rate 88%) at the age of 15 to 16 years, and these were converted into body mass index (BMI), which was calculated as weight (kg) divided by height squared (m^2^). If the measured values for height and weight were missing, we used self-reported values (for 12% of adolescents). Normal weight was defined as <23.90 kg/m^2^ for boys and <24 kg/m^2^ for girls, overweight as 23.90–28.88 kg/m^2^ for boys and 24–29.43 kg/m^2^ for girls, and obesity as >28.88 kg/m^2^ for boys and >29.43 kg/m^2^ for girls. These scales were assessed using the International Obesity Task Force age-specific cut-off points for BMI, corresponding to a BMI of 25 and 30 for adults [42].

#### Psychosocial variables

The measure for screening the emotional and behavioral problems of adolescents during the preceding six months was the Youth Self-Report (YSR) questionnaire [38]. The documentation of the construct, criterion-related and content validity, and the reliability of the YSR, based on the test-retest correlations, have proven to be good [38]. The reliabilities of the YSR scales ranged between 0.69 and 0.83, with aggressive behavior highest and social problems lowest when measured with Cronbach's alpha coefficient, which is used to test the internal consistency of the scale [38]. In the questionnaire, the individuals rated themselves in each of 105 items on a scale of 0–2: 0 = not true, 1 = somewhat or sometimes true and 2 = very true or often true. The items were summed and scored on eight syndrome subscales: (1) anxious/depressed symptoms, (2) withdrawn/depressed symptoms, (3) somatic complaints, (4) social problems, (5) thought problems, (6) attention problems, (7) rule-breaking behavior, and (8) aggressive behavior. In the present study, broadband scales are termed: “internalizing”, made up of anxious/depressed symptoms, withdrawn/depressed symptoms and somatic complaints, and “externalizing syndromes” represent rule-breaking and aggressive behaviors. Subscales 4–6 were considered as “other symptoms” and they were not included in either internalizing or externalizing scales. Adolescents were trichotomized as normal range, borderline range and clinical range groups consistent with the recommended cut-off limits [43]. For analysis, the two last groups were combined to form the “problem range” (=above 82nd percentile). The analysis was restricted to adolescents with more than eight missing responses to the YSR (excluding open-ended and socially desirable items, altogether 15 items). In other cases, the mean item value of the particular scale was used for substituting the missing values.

#### Assessment of socio-economic status

The socio-economic status of family is likely to influence on health behaviors and psychosocial health. Information on status was assessed from the questionnaire sent to the parents, and the father’s response was prioritized. The response was based on a five-item work and education questionnaire: (1) higher clerical employees; (2) self-employed; (3) lower clerical employees; (4) workers; and (5) students, pensioners, unemployed or unknown. The correlations of socio-economic status and LCA variables are presented in Table 1.

**Table 1:** Spearman’s correlation coefficients of psychosocial and lifestyle factors and socio-economic status of family

	** Boys**		** Girls**	
	**Correlation coefficient**	**P value**	**Correlation coefficient**	**P value**
Internalizing problems	0.01	0.551	−0.01	0.785
Externalizing problems	0.06	0.003	0.02	0.399
Physical activity, h/day	−0.03	0.101	−0.06	0.001
Sitting time, h/day	0.11	<0.001	0.10	<0.001
Sleeping, h/day	0.00	0.939	0.02	0.304
BMI, kg/m^2^	−0.01	0.508	0.01	0.708
Smoking	0.11	<0.001	0.08	<0.001
BMI = body mass index				

### Follow-up study

Physical activity level and smoking were assessed in exactly the same way at 18 years to that at 16 years. Physical activity was categorized as <2 hours of MVPA/week and ≥2 hours of MVPA/week, and smoking as non-smoking and smoking. BMI was based on reported values on questionnaire and overweight participants were defined as those with a BMI of ≥25 kg/m^2^, as recommended earlier [42].

### Data analysis

Data analysis was conducted with Latent Class Analysis (LCA) using M-plus version 6.11. LCA [26,27] is a statistical model that estimates the number of latent homogenous clusters existing within a heterogeneous population. It assesses the pattern of observed responses and identifies the size and characteristics of each group. The LCA model assumes that the relationships among a set of observed variables are explained by an unmeasured “latent” categorical variable with discrete clusters [44].

The fundamental procedure in LCA is to increase the cluster number until the most parsimonious model for the used data is found. In this work, we assessed LCA models with one to seven clusters. To determine the best-fitting cluster solution, we calculated the Akaike information criterion (AIC; [45]), the Bayesian information criterion (BIC; [46]), and the sample-size adjusted BIC (SSABIC; [47]) and used them to measure goodness-of-fit. According to the criteria of these statistical fit indices, the lower the values, the better the model fit [48].

We also used Lo-Mendell-Rubin's adjusted likelihood ratio test (LRT; [49]), average latent class cluster classification accuracy, and entropy measures for identifying the optimal number of clusters. The differences between models with differing numbers of clusters were exposed by the LRT statistics, where a low p-value (<0.05) indicated that the qualified model fits the used data better than the model with one less cluster. Entropy is a measure of uncertainty in the classification, its values ranging from 0 to 1 [50]. Higher values indicated greater precision of delineation of clusters, while lower values suggested an unclear classification of individuals into the latent class clusters.

In addition to statistical fit indices, the interpretability of the classification, the conceptual meaningfulness of the models, and the sizes of the subgroups were taken into account in the selection of the most appropriate cluster solution [29,37].

## Results

### Estimation of number of latent clusters

Of the tested LCA models (Table 2), the four-cluster solution fit the data best in both sexes. Among boys, this model had the lowest BIC value, which has been preferred in the cluster number selection by previous research [51]. In addition, the conceptual meaningfulness and sizes of the clusters (the five-cluster model included one cluster with only 52 participants; data not shown) stressed the superiority of four-cluster solutions over the five-cluster model. Among girls, the lowest BIC value, and low AIC and SSABIC values, as well as high entropy and the LRT of the four-cluster model being superior to the three-cluster solution, led to the choosing of the model with four clusters.

**Table 2:** Fit statistics for a one-class model through to a seven class-model by sex

**Number of classes in the model**	**Fit statistics**				
	**AIC**	**BIC**	**SSABIC**	**Entropy**	**LRT**
Boys					
1-Class model	38310.975	38384.202	38346.073	N/A	N/A
2-Class model	37755.683	37902.138	37825.879	0.811	0.1190
3-Class model	37390.284	37609.967	37495.578	0.784	0.3129
4-Class model	37275.750	37568.660	37416.142	0.644	0.0008
5-Class model	37236.918	37603.055	37412.408	0.833	<0.001
6-Class model	37209.182	37648.546	37419.770	0.679	0.2307
7-Class model	37140.859	37653.450	37386.545	0.822	1.0000
Girls					
1-Class model	26843.382	26908.612	26870.482	N/A	N/A
2-Class model	25793.163	25935.439	25862.357	0.654	<0.0001
3-Class model	25706.917	25917.237	25809.203	0.634	0.0141
4-Class model	25328.046	25606.412	25463.425	0.804	0.0050
5-Class model	25295.221	25641.632	25463.692	0.707	0.4722
6-Class model	25255.253	25669.709	25456.817	0.762	0.6387
7-Class model	25283.651	25766.152	25518.307	0.824	0.1721

AIC = Akaike Information Criteria, BIC = Bayesian Information Criteria, SSABIC = Sample Size Adjusted Bayesian Information Criteria.

LRT = *p*-value for the Lo-Mendell-Rubin Likelihood Ratio Test.

### Adolescent’characteristics

At 16 years, 11% of boys and 20% of girls had internalizing problems, while 16% of boys and 26% of girls suffered from externalizing problems (Tables 3 and 4). As regards physical activity, 31% of boys and 41% of girls exercised for a maximum of one hour per week. 35% of boys reported sitting for more than eight hours per day, while the mean value of total sitting time was 6.3 hours among girls. Moreover, 17% of boys and 25% of girls did not meet the recommendation of over eight hours of sleep per day, the mean values of BMI were 21.1 for boys 21.2 for girls, and 8% of both boys and girls had smoked over 1.0 pack-years by the age of 16.

**Table 3:** Prevalence rates and proportions or mean values of each psychosocial and lifestyle variables for the four clusters among boys

	**All**	**Externalizing behavior**	**Sedentary**	**Obese**	**Reference**
Prevalence	100%	14.3%	26.8%	7.8%	51.0%
Internalizing problems^a^	0.11	0.31	0.15	0.12	0.04
Externalizing problems^a^	0.16	1.00	0.00	0.20	0.00
Physical activity^a^, h/week					
<2	0.31	0.34	0.50	0.43	0.18
2–3	0.30	0.19	0.30	0.29	0.21
>3	0.39	0.48	0.20	0.28	0.60
Sitting time^a^, h/day					
< 4.1 h	0.22	0.17	0.07	0.14	0.33
4.1–7.9 h	0.43	0.43	0.25	0.37	0.54
> 7.9 h	0.35	0.41	0.68	0.48	0.13
Sleeping time^a^, h/day					
<8 h	0.17	0.27	0.39	0.25	0.01
8–9 h	0.67	0.54	0.47	0.60	0.82
>9 h	0.17	0.19	0.15	0.15	0.17
BMI^a,b^, kg/m^2^					
mean value (CI)	21.1 (21.0–21.2)	20.6 (20.4–20.9)	20.1 (20.0–20.3)	29.7 (29.3–30.2)	20.5 (20.4–20.6)
Smoking^a^					
non-smoker	0.84	0.54	0.79	0.86	0.94
0.1–1.0 pack-years	0.08	0.23	0.09	0.07	0.04
>1.0 pack-years	0.08	0.23	0.12	0.07	0.02

^a^p < 0.001.

^b^Continuous variables used.

CI = confidence interval.

BMI = body mass index.

**Table 4:** Prevalence rates and proportions or mean values of each psychosocial and lifestyle variables for the four clusters among girls

	**All**	**Externalizing behavior**	**Multiple risk behaviors**	**Obese**	**Reference**
Prevalence	100%	15.0%	11.8%	6.7%	66.5%
Internalizing problems^a^	0.20	0.36	0.40	0.18	0.12
Externalizing problems^a^	0.26	1.00	0.80	0.22	0.00
Physical activity^a^, h/week					
<2	0.41	0.31	0.73	0.52	0.36
2–3	0.30	0.30	0.19	0.30	0.32
>3	0.29	0.39	0.08	0.18	0.32
Sitting time^a,b^, h/day					
mean value (CI)	6.3 (6.2–6.4)	5.4 (5.2–5.6)	9.5 (9.1–9.9)	7.3 (6.8–7.8)	5.9 (5.7–6.0)
Sleeping time^a^, h/day					
<8 h	0.25	0.24	0.53	0.30	0.20
8–9 h	0.65	0.65	0.32	0.55	0.72
>9 h	0.10	0.11	0.15	0.16	0.09
BMI^a,b^, kg/m^2^					
mean value (CI)	21.2 (21.1–21.3)	20.6 (20.4–20.8)	20.9 (20.7–21.2)	29.9 (29.4–30.5)	20.5 (20.4–20.6)
Smoking^a^					
non-smoker	0.80	0.68	0.19	0.80	0.93
0.1–1.0 pack-years	0.12	0.29	0.26	0.13	0.06

^a^p < 0.001.

^b^Continuous variables used.

CI = confidence interval.

BMI = body mass index.

### Characteristics of the four-cluster model

Boys in Cluster 1 (prevalence rate 14.3%; hereafter referred to as *Externalizing behavior*) had the highest likelihoods of externalizing (1.00) and internalizing (0.31) problems, and smoking of all the clusters, but the probability of being physically active was also considerably high (0.48). In Cluster 2 (26.8%; hereafter referred to as *Sedentary*) the probabilities of physical inactivity (0.50), sitting over eight hours per day (0.68), and having poor sleeping times (0.39) were the highest. In Cluster 3 (7.8%; hereafter referred to as *Obese*), boys had a very high BMI, relatively high probabilities of physical inactivity (0.43) and a sitting time of over eight hours per day (0.48). In Cluster 4 (51%; hereafter referred to as *Reference*) the likelihoods of sleeping and exercising were the highest, and the probabilities of internalizing symptoms, sitting over eight hours per day, and having problems with weight and smoking were the lowest.

Girls in Cluster 1 (15%; hereafter referred to as *Externalizing behavior*) had externalizing problems (1.00) and a relatively high likelihood of internalizing problems (0.36). However, these girls also had the highest probability of physical activity, and the least sitting time of all the subgroups. In Cluster 2 (11.8%; hereafter referred to as *Multiple risk behaviors*) girls were the most likely to have internalizing problems (0.40), particularly somatic complaints (data not shown), low levels of physical activity (0.73), long sitting times per day, short sleeping times (0.53), and were most likely to smoke (0.81). A great probability of externalizing problems, especially rule-breaking behavior (data not shown), was also observed in this subgroup (0.80). In Cluster 3 (6.7%; hereafter referred to as *Obese*) girls had a very high BMI. The likelihood of physical inactivity and average sitting time were also comparably high (0.52 and 7.3 h/day). In Cluster 4 (66.5%; hereafter referred to as *Reference*) girls had the highest probability of sleeping sufficiently, the lowest likelihoods of smoking, with higher BMI or having internalizing problems, and a comparably high probability of exercising. The distribution of psychosocial and lifestyle factors in each of the latent class clusters for the four-cluster model are presented as stratified by sex in Tables 3 and 4.

### Prevalence of physical inactivity, overweight and smoking within the clusters at baseline and follow-up

#### Boys

**Figure 1:**
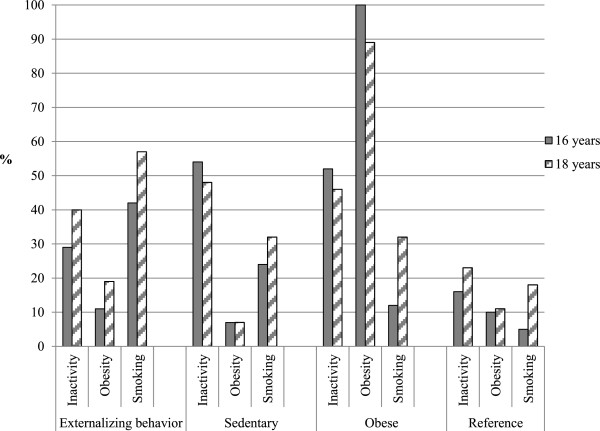
Proportions of inactive, overweight, and smoking boys within the clusters at 16 and 18 years.

At baseline, in the *Externalizing behavior* cluster, a third exercised for less than two hours, a few were overweight, and smoking was prevalent (42%) (Figure 1). At 18 years, physical inactivity and overweight showed a slightly higher prevalence than at 16 years (40% and 19%). The proportion of smokers increased significantly during the follow-up, reaching nearly 60% at 18 years. In the *Sedentary* cluster, physical inactivity was high (54%) and overweight and smoking at a low level (7% and 24%) at 16 years. At follow-up, the prevalence of physical inactivity stayed high and overweight low. Smoking occurred among a third of boys. In the *Obese* cluster physical inactivity was highly prevalent (52%) and overweight extremely prevalent (100%), whereas only some smoked at baseline. At 18 years, physical inactivity remained common. The prevalence of overweight was slightly lower and the amount of smokers higher than at 16 years. However, overweight still remained extremely prevalent and only a third of boys smoked at 18 years. In the *Reference* cluster, each behavior was uncommon. In the follow-up, physical inactivity and smoking increased, but the proportions were still low (23% and 18%).

#### Girls

**Figure 2:**
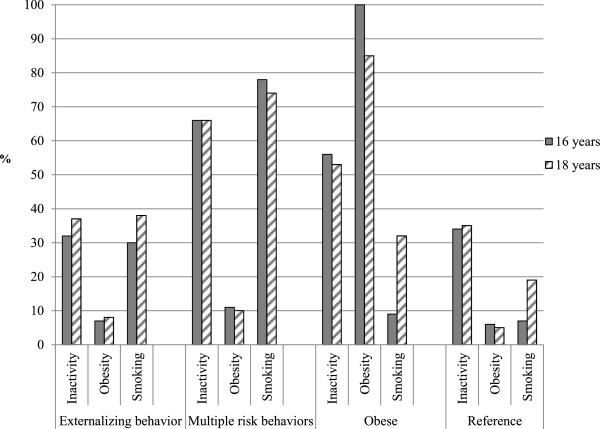
Proportions of inactive, overweight, and smoking girls within the clusters at 16 and 18 years.

At baseline, in the *Externalizing behavior* cluster, nearly a third was inactive, a few were overweight, and smoking occurred among 30% of girls (Figure 2). At follow-up, the proportions did not significantly differ from those at 16 years. In the *Multiple risk behaviors* cluster, physical inactivity was highly and smoking extremely prevalent (66% and 78%), whereas nearly none were overweight at 16 years. At follow-up, the prevalence of physical inactivity and smoking remained at significant levels (66% and 74%) and that of overweight at a low level. In the *Obese* cluster, physical inactivity was common (52%) and overweight the most prevalent (100%), but almost none smoked. At 18 years, the proportion of inactive girls was similar to that at 16 years. Overweight was slightly less prevalent and smoking more common at 18 years than at 16 years, but the prevalence of overweight still remained extremely high (85%) and the amount of smokers relatively low at 18 years (32%). In the *Reference* cluster, a third was physically inactive, and a few were overweight and smoked. At follow-up, physical inactivity level and the amount of overweight adolescents remained similar to that at 16 years, while the proportion of smokers increased between the ages of 16 and 18. However, smoking remained at a low level at 18 years (19%).

## Discussion

### Main findings

Health-related behaviors and psychosocial symptoms divided adolescents into distinct subgroups in which adverse lifestyle patterns, including low levels of physical activity, high BMI and smoking tended to persist between the ages of 16 and 18. Smoking co-occurred with psychosocial symptoms in both genders. Among girls, several unhealthy behaviors and psychosocial symptoms accumulated, whereas inactive boys had more sleeping problems than the others. Adolescents with a high BMI emerged as a group of their own in both sexes. Unhealthy behaviors and psychosocial problems were uncommon among one half of boys and two-thirds of girls. Correlations between socio-economic status and adverse lifestyle, emotional and behavioral problems were low.

### Some relations to existing literature

In the current work, adolescents with emotional and behavioral symptoms smoked the most, and this unhealthy behavior continued at the age of 18, and may do so later on [52]. A wide array of studies have illustrated significant associations of emotional and behavioral problems [24,43,53] and the co-occurrence of both symptoms [54,55] with cigarette use. Whereas Colder et al. [54] and Miller-Johnson et al. [55] established an association between high levels of co-occurring psychosocial problems and smoking, we observed that psychosocial symptoms occur at differing rates, with externalizing problems being more prevalent. Youth with co-occurring psychosocial symptoms tend to have a greater number of friends involved in risky lifestyles [56], and this is likely to endanger them to risk-taking behavior, which may include smoking. On the other hand, cigarettes may serve as self-medication for improving attention or managing stress.

Surprisingly, our analyses also showed that psychosocial symptoms overlap with a higher level of physical activity among boys. A similar finding was also observed among girls, but only among some of those who had psychosocial problems. Even though it is in contrast with common assumptions [23,25], positive associations between higher physical activity and psychosocial problems also exist [57-59]. It seems that only certain subscales of psychosocial problems, e.g. aggressive behavior [57,58], and types of sports, e.g. team sports with aggressive characteristics [59], are relevant to the existence of the relation. However, we found no explanatory differences in subscales of psychosocial symptoms or in sporting activities between the subgroups (data not shown). Perhaps disparities in the smoking behavior determine co-occurrence among girls.

Nearly 12% of the girls in our study had problems with psychosocial or lifestyle issues including physical inactivity, sedentary behavior, and short sleeping times. The accumulation of multiple risk factors is a prevalent phenomenon among adolescents [60,61], and associations of female gender [61,62], depressiveness [63], low self-esteem [64], high anxiety scores [60], and smoking cigarettes [21] with the presence of the multiple health-related risk factors previously acknowledged lend support to our observations. It is likely that some underlying factors, perhaps related to the social environment, for example, the habits of family and friends [60,64], have influenced the concentration of several behaviors. Moreover, a greater number or heightened levels of risk factors may have occurred among these girls.

Obtaining sufficient levels of sleeping times constituted a notable problem for the most sedentary and physically inactive boys, which is in line with some [8,65], but not all studies [66] investigating the associations between these factors. An LCA study by Laska et al. [35] found that inadequate physical activity clustered with inadequate sleep patterns, but additional knowledge of the patternings of all three behaviors seems to be limited. One possible explanation for our results may be that watching television, using computers or perhaps doing homework in the late evening results in delayed bedtimes and frequent difficulties in falling asleep [67], which in turn limits the opportunity to get sufficient sleep. This may lead to daytime sleepiness [68], especially during school days, and also have an effect on exercise behavior. In contrast, regular physical activity tends to improve sleep quality, which may also explain the observed relation [69]. Surprisingly, short sleeping time did not coincide with psychosocial symptoms among boys, which suggests that lifestyle factors might be more relevant in boy’ sleeping behavior.

Among both boys and girls, a group with a very high BMI emerged. These adolescents had comparably low levels of physical activity and long sitting times, but other unhealthy behaviors and psychosocial symptoms appeared to be rare. This is a surprising finding, since one could expect that the most inactive adolescents would have the highest BMI [31,70] or at least that weight problems would exist among several groups. However, moderate patterns of physical activity and sedentary behavior have also been linked to increased BMI [70]. In addition, it is likely that these adolescent’ eating behaviors, not evaluated in our study, differ from the others’.

### Strengths and weaknesses

To the best of the author’ knowledge, the current work is the first to apply LCA to patterning of both psychosocial and lifestyle factors in a sample of adolescents. The data was based on a large birth cohort, which is also definitely another strength of this study. Despite the follow-up population being a subcohort of the original cohort, it can be considered a representative sample [71].

A few limitations of this study should also be taken into account. Firstly, our results relied on self-report values, except BMI at 16 years. Participants may have under- or over-reported their behaviors on account of social desirability, for example, which may have led to bias. However, previous studies of adolescents have suggested that self-administered questionnaires are reliable methods for assessing adolescent’psychosocial and lifestyle behaviors [7,38,72]. Compared to self-reported values, accelerometer-derived physical activity/inactivity would have been more accurate method to investigate activity patterns but in a large population-based study as the current work it would have been difficult to conduct. Secondly, a larger proportion of boys than girls belonging to the NFBC 1986 did not fill in the YSR questionnaire properly or did not respond at all (32% of boys vs. 21% of girls; [36]). Non-respondent males were found to have slightly more problems than those who replied [36]. Thus, we may have underestimated the psychosocial problems among the boys. Thirdly, we were unable to study the persistence of sleep and psychosocial symptoms, as we did not inquire about sleeping or measure psychosocial problems with the YRS questionnaire in the follow-up. Finally, the follow-up period was quite short to fully understand the persistence of adverse health-related behaviors.

## Conclusions

Our study identified various subgroups of 16-year-old adolescents, in which lifestyle risks and psychosocial burden concentrate and tend to persist at least at the age of 18. The provided information is likely to be useful for tailoring more inclusive health promotion programs for youth [73]. Still, further research is needed to evaluate the efficacy of preventions targeted to these groups of adolescents, as well as the early predictors of memberships of the observed clusters. The association between the clusters and subsequent health is also a relevant study question.

## Competing interests

The authors declare that they have no competing interests.

## Author’ contributions

EH conceived of the study, participated in its design, and drafted the manuscript; JR performed the statistical analyses; MP, ST, JA, and JK designed the study’s analytic strategy, helped interpret the results, and supervised the whole process. All the authors have seen and approved the final version.

## Pre-publication history

The pre-publication history for this paper can be accessed here:

http://www.biomedcentral.com/1471-2458/14/542/prepub
